# A peek into cancer-associated fibroblasts: origins, functions and translational impact

**DOI:** 10.1242/dmm.029447

**Published:** 2018-04-19

**Authors:** Valerie S. LeBleu, Raghu Kalluri

**Affiliations:** Department of Cancer Biology, Metastasis Research Center, University of Texas MD Anderson Cancer Center, Houston, TX 77005, USA

**Keywords:** Cancer, Fibroblasts, Heterogeneity

## Abstract

In malignant tumors, cancer cells adapt to grow within their host tissue. As a cancer progresses, an accompanying host stromal response evolves within and around the nascent tumor. Among the host stromal constituents associated with the tumor are cancer-associated fibroblasts, a highly abundant and heterogeneous population of cells of mesenchymal lineage. Although it is known that fibroblasts are present from the tumor's inception to the end-stage metastatic spread, their precise functional role in cancer is not fully understood. It has been suggested that cancer-associated fibroblasts play a key role in modulating the behavior of cancer cells, in part by promoting tumor growth, but evolving data also argue for their antitumor actions. Taken together, this suggests a putative bimodal function for cancer-associated fibroblasts in oncogenesis. As illustrated in this Review and its accompanying poster, cancer-associated fibroblasts are a dynamic component of the tumor microenvironment that orchestrates the interplay between the cancer cells and the host stromal response. Understanding the complexity of the relationship between cancer cells and cancer-associated fibroblasts could offer insights into the regulation of tumor progression and control of cancer.

## Abstract (precis)

**Summary:** Cancer-associated fibroblasts constitute a functionally heterogeneous mesenchymal cell population in the tumor microenvironment. This ‘At a glance’ article reviews their origin and their pro- and antitumor properties.

## Introduction

The tumor microenvironment (TME; see Glossary, Box 1) contains a heterogeneous population of cells with overlapping or opposing functions that impact tumor growth and cancer progression (Augsten, 2014; Gascard and Tlsty, 2016; Ishii et al., 2015; Kalluri, 2016). A dominant cell type found in solid tumor lesions is the mesenchymal or fibroblastic cell type, also referred to as cancer-associated fibroblasts (CAFs). CAFs are a ‘family’ or ‘group’ of cells that exhibit mesenchymal-like features and are likely mesoderm derived (Box 1). They are found in the vicinity or in direct contact with neoplastic cells (Kalluri, 2016), and are often the dominant cell type within a solid tumor mass (Augsten, 2014; Kalluri, 2016). In contrast, normal or tissue resident fibroblasts represent a more discrete proportion of cells that reside in a given organ. These are likely quiescent or resting cells that are capable of responding to extrinsic cues, such as growth factors, cytokines and mechanical stress, to become activated (Kalluri, 2016; Rasanen and Vaheri, 2010; Shiga et al., 2015). The parenchymal injury associated with a nascent and growing tumor is an example of such a cue that can lead to the activation of normal fibroblasts, thereby giving rise, at least in part, to the CAFs expanding in the tumor (Alexander and Cukierman, 2016; Kalluri, 2016).

**Figure:**
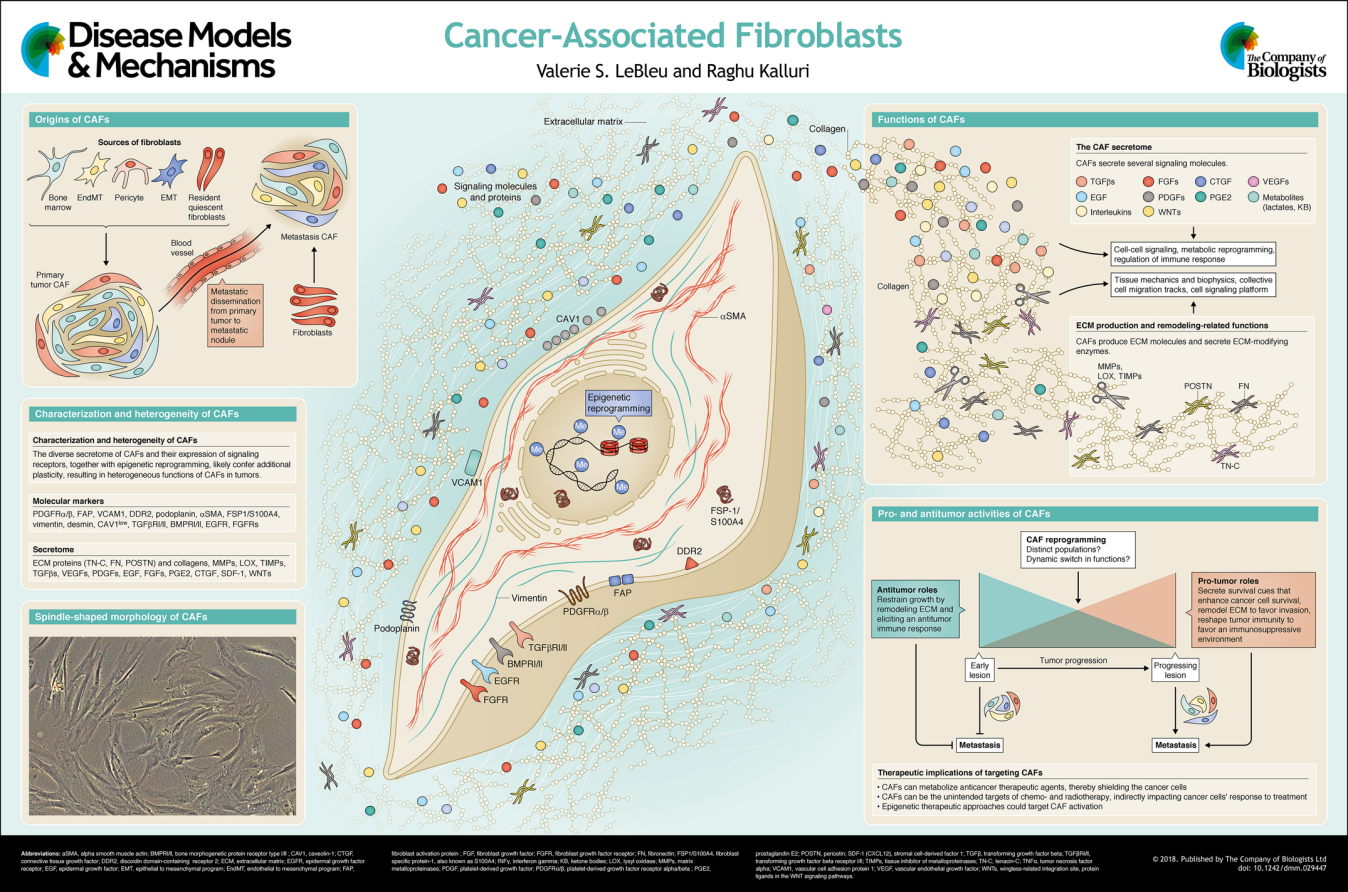

**Box 1. Glossary**

**Desmoplastic reaction:** Secondary to an initial tissue injury, it is the collective response of stromal cells, including activated fibroblasts and recruited immune cells, in generating scar tissue.

**Endothelial-to-mesenchymal transition (EndMT):** a cellular program wherein endothelial cells lose some of their features and gain mesenchymal-like characteristics (reviewed in Potenta et al., 2008; Yu et al., 2014a).

**Epithelial-to-mesenchymal transition (EMT):** a cellular program wherein epithelial cells lose some of their features and gain mesenchymal-like characteristics (reviewed in Kalluri, 2009; Kalluri and Weinberg, 2009).

**Extracellular matrix (ECM):** the secreted fibrous proteins and proteoglycan assembling into a supportive network that enables tissue organization, cellular adhesion, proliferation and migration (ECM in cancer reviewed in Lu et al., 2012).

**Mesenchymal stromal cells (MSCs):** historically defining a population of bone marrow-derived cells that present as adherent, fibroblast-like cells following their isolation. This population of cells may include cells with multipotent properties, also referred to as mesenchymal stem cells.

**Mesoderm:** the middle germ layer in the developing embryo that emerges during gastrulation and is in between the other two germ layers, namely, the ectoderm and endoderm.

**Metronomic chemotherapy:** a low-dose, continuous chemotherapeutic regimen aimed to target tumor angiogenesis together with cancer cells.

**Paracrine signaling:** a form of communication between cells where signaling factors (such as growth factors) are secreted by a cell to elicit a change in the nearby recipient cell that responded to the signaling factor.

**Pericytes:** or perivascular cells, the cells lining the abluminal (outer) surface of microvessels (reviewed in Armulik et al., 2011).

**Tumor microenvironment (TME):** noncancer cells and ECM found in a tumor, which includes CAFs, blood vessels and immune cells (reviewed in Balkwill et al., 2012; Chen et al., 2015; Quail and Joyce, 2013).

The appellation ‘CAFs’ is often used as an umbrella term to define a complex population of dynamically heterogeneous mesenchymal cells, with functions that are likely distinct from those of resident tissue fibroblasts (Cortez et al., 2014; Ishii et al., 2015; Kalluri, 2016). The majority of studies describe CAFs as producers of cytokines, chemokines, metabolites, enzymes and extracellular matrix (ECM; Box 1) molecules that fuel the growth of cancer cells (Kalluri, 2016) (see poster). However, the net outcome of the biosynthetic secretome of CAFs could limit, just as it could promote, cancer progression (Augsten, 2014; Ishii et al., 2015; Kalluri, 2016), as will be discussed in more detail below.

The origin and functions of CAFs are likely as diverse as the markers used for their identification (see poster), yielding a complex picture of their composition, dynamic lineage evolution, and functional roles at various stages of cancer progression (Augsten, 2014; Cortez et al., 2014; Kalluri, 2016; Madar et al., 2013; Öhlund et al., 2014). Here, we summarize the complex features of CAFs to inform on their origin, activation, accumulation, heterogeneity and function. Much like the complexity of the tumor immune response, CAFs also exhibit complex tumor-associated phenotypes, suggestive of their distinct functions (Augsten, 2014; Cirri and Chiarugi, 2012; Ishii et al., 2015; Kalluri, 2016; Luo et al., 2015; Madar et al., 2013; Öhlund et al., 2014). We also discuss the distinct functions of CAFs in promoting and restraining cancer. We summarize their roles in cancer progression, which are wide-ranging and include the production of ECM components and remodeling enzymes, as well as the secretion of metabolites, cytokines, and growth factors that signal to cancer cells (see poster) and influence tumor angiogenesis and immune infiltration. We also discuss their less-known cancer-restraining functions, which are predominantly associated with the regulation of early antitumor response and tumor metabolism. CAFs also express a number of signaling receptors that are engaged in maintaining or changing the CAF phenotypes during cancer progression. These receptors might also be involved in integrating signals from various cell types within the TME, thus further influencing the functioning of CAFs, and are discussed both in this article and in the accompanying poster.

## Origins and characteristics of CAFs

A significant proportion of CAFs likely emerge from a mesoderm-derived precursor cell, although the precise origin of all CAFs in a given tumor bed is still not fully understood and is likely mixed (Madar et al., 2013) (see poster). Gaining further insights about the origin of CAFs could offer novel understanding of their plasticity, identifying markers, signaling cues that lead to their activation, and means to target their pro-tumorigenic and/or enhance their antitumorigenic functions. When a cancer arises in the adult organ, the dominant niche likely includes the expansion of quiescent fibroblasts residing in the host tissue in response to the injury caused by the developing neoplasm (reviewed in Kalluri, 2016). Additionally, CAFs can be recruited to the tumor from a distant source, such as the bone marrow (reviewed in Kalluri, 2016; Shiga et al., 2015). The trans-differentiation of pericytes (Box 1), endothelial and epithelial cells can also give rise to a CAF-like hybrid cell population when the latter two undergo the endothelial-to-mesenchymal transition (EndMT; Box 1) (Potenta et al., 2008) and the epithelial-to-mesenchymal transition (EMT; Box 1) (Kalluri and Weinberg, 2009) programs, respectively. The notion that CAFs can, similarly to cancer cells, disseminate into the circulation and to distant metastatic sites, suggests that CAFs have additional complex roles in metastasis (Cirri and Chiarugi, 2012; De Wever et al., 2014).

Despite the technical advances in genetic lineage tracing (also known as fate mapping) and in fluorescent tagging to elucidate the origin(s) of CAFs in tumor-bearing mice (LeBleu et al., 2013; O'Connell et al., 2011; Ozdemir et al., 2014), the inherent difficulty in clearly identifying their biological origin is due to the lack of specific markers for fibroblasts. In microscopic analyses of tissue sections, CAFs can be identified based on their spindle shape and elongated cytoplasmic processes (Hematti, 2012; Ishii et al., 2015; Kalluri, 2016). Notably, they were experimentally found to be easy to adapt to tissue culture conditions, and expand *in vitro* as spindle-shaped cells (see poster). They can be distinguished from other cell types within the tumor by exclusion criteria defined by their morphological features and a lack of expression of nonmesenchymal markers, such as those expressed by endothelial, epithelial, immune and neuronal cells; and based on inclusion criteria defined by the expression of a slew of posited mesenchymal markers, although none of these has absolute specificity (Gascard and Tlsty, 2016; Kalluri, 2016; Rasanen and Vaheri, 2010; Shiga et al., 2015).

So far, researchers have identified an exhaustive list of candidate markers for CAFs (Ishii et al., 2015; Kalluri, 2016), noting that their relative expression and abundance, and distinct overlapping expression patterns in different tissue types (Liao et al., 2018; Roswall and Pietras, 2012; Sugimoto et al., 2006) all contribute to the challenge in determining the biological origin of CAFs in growing tumors. Some of the most commonly utilized markers, possibly due to their overlapping expression amongst a large population of CAFs, are discussed below and listed in the second panel and the centered schematic of the poster. Although this is still an ongoing area of investigation in many laboratories, distinct tumor types can present with different abundance and overlap in a given set of CAF markers. The abundance of a given CAF marker in a tumor type might represent features of activation of the dominant type of resident fibroblasts in the impacted tissue. For example, αSMA^+^ CAFs (see Abbreviations, Box 2) are dominantly found in pancreatic carcinoma and might reflect the activation of resident stellate cells (Ferdek and Jakubowska, 2017; Ozdemir et al., 2014), whereas PDGFRα^+^ CAFs (Box 2) in melanoma might reflect the activation and expansion of resident dermal fibroblasts that express this marker (Anderberg et al., 2009; Lynch and Watt, 2018). Comparative analyses of Rip1Tag2 pancreatic carcinoma and 4T1 orthotopic breast carcinoma in mice showed distinct overlap of CAF markers, with as many as 43.5% of FSP1/S100A4^+^ (Box 2) fibroblasts showing co-expression of αSMA in pancreatic carcinoma, whereas only 10.9% of FSP1/S100A4^+^ fibroblasts showed co-expression of αSMA in breast carcinoma (Sugimoto et al., 2006).

**Box 2. Abbreviations**

αSMA: alpha smooth muscle actin
BMPRI/II: bone morphogenetic protein receptor type I/II
CAV1: caveolin-1
CSL: CBF1/Su(H)/Lag-1 transcription factor complex
CTGF: connective tissue growth factor
DDR2: discoidin domain-containing receptor 2
EGFR: epidermal growth factor receptor
FAP: fibroblast activation protein
FGF2: fibroblast growth factor 2
FGFR: fibroblast growth factor receptor
FSP1/S100A4: fibroblast-specific protein 1, also known as S100A4
IL-10: interleukin 10
IL-6: interleukin 6
INFγ: interferon gamma
LIF: leukemia inhibitory factor
LOX: lysyl oxidase
LOXL1: lysyl oxidase-like 1
MMPs: matrix metalloproteinases
PDGF: platelet-derived growth factor
PDGFRα/β: platelet-derived growth factor receptor alpha/beta
PGE2: prostaglandin E2
SDF-1 (CXCL12): stromal cell-derived factor 1
SHH: sonic hedgehog
TGFβ: transforming growth factor beta
TGFβRI/II: transforming growth factor beta receptor I/II
TIMPs: tissue inhibitors of metalloproteinases
TNFα: tumor necrosis factor alpha
VCAM1: vascular cell adhesion protein 1
VEGF: vascular endothelial growth factor
WNTs: wingless-related integration site, protein ligands in the WNT signaling pathways
CTLA-4 (CD152): cytotoxic T-lymphocyte protein 4
PD-L1: programmed death-ligand 1

To define and identify the origin of fibroblasts, it is crucial to consider that CAFs are ‘activated fibroblasts’, which, in contrast to nonactivated (quiescent) tissue-resident fibroblasts, are an expanding population of cells that either proliferates *in situ* or is recruited to the tumor (Kalluri, 2016; Ozdemir et al., 2014; Rasanen and Vaheri, 2010). The key features of CAFs, distinguishing them from quiescent fibroblasts, include metabolic adaptations to support their need for enhanced proliferation and biosynthetic activities, such as production of ECM components and enzymes to remodel the ECM, growth factors and cytokines (Alexander and Cukierman, 2016; Erez et al., 2010; Han et al., 2015; Harper and Sainson, 2014; Kalluri, 2016; Marsh et al., 2013; Öhlund et al., 2014; Rasanen and Vaheri, 2010; Raz and Erez, 2013; Wu et al., 2017). Although the distinct functions of CAFs could inform on their origins, these functions might dynamically shift during cancer progression, likely reflecting the flexibility of CAFs in adapting to a changing (tumor) microenvironment.

## Activation and heterogeneity of CAFs

As their appellation infers, CAFs are defined by their association with cancer cells within a tumor. In carcinomas, their biology is generally studied in relation to the biology of genetically aberrant neoplastic epithelial (cancer) cells. It is therefore critical to appreciate that CAFs emerge as part of the host's response to epithelial injury caused by the growing tumor (Ishii et al., 2015; Kalluri, 2016). The initial recruitment of CAFs to the nascent neoplastic lesions might thus reflect their role in the early antitumor response (Kalluri, 2016; Marsh et al., 2013). In wounds, activated fibroblasts accumulate and facilitate many aspects of the tissue remodeling cascade to initiate the repair process and to control and prevent further tissue damage (Bainbridge, 2013; Kalluri, 2016; Öhlund et al., 2014). Activated fibroblasts also induce an intrinsic program, likely influenced by other cells, to limit an excessive scarring response, which would otherwise further injure the tissue (Duffield et al., 2013; Kalluri, 2009; Klingberg et al., 2013; Zeisberg and Kalluri, 2013). An example of the detrimental action(s) of fibroblasts in response to epithelial damage is organ fibrosis, a condition associated with unabated fibroblast activation that results in chronic inflammation and impaired functional regeneration of the impacted tissue (Duffield et al., 2013; Zeisberg and Kalluri, 2013). The mechanisms underlying this unabated activation of fibroblasts remain largely unknown, although epigenetic reprogramming might, at least in part, contribute to this sustained activated state (Albrengues et al., 2015; Bechtel et al., 2010; Zeisberg and Zeisberg, 2013). For example, hypermethylation of the *RASAL1* promoter leads to its transcriptional suppression, increased Ras-GTP activity and perpetuated activation of fibroblasts, which is promoted in renal fibrosis (Bechtel et al., 2010). Interestingly, a global hypomethylation of the genomes of CAFs was also reported (Jiang et al., 2008), possibly driving the upregulation of genes associated with the CAF secretome. Moreover, biological aging or senescence of fibroblasts are associated with the secretion of various pro-tumorigenic factors that can contribute to CAF activation in oncogenesis. The concomitant downregulation of the NOTCH protein effector CSL (Box 2) and p53 overcomes the senescence failsafe mechanism and enables CAF activation and proliferation (Procopio et al., 2015).

We speculate that fibroblasts become activated during the initial stages of oncogenesis, giving rise to CAFs, which then remodel the tumor microenvironment to elicit tissue repair, thereby possibly exerting antitumor functions. However, as the tumor grows, this repair process might, in turn, promote tumor growth, as cancer cells utilize the CAF-secreted growth factors to facilitate their own survival and proliferation. A precise tipping point between the functions of CAFs in tissue repair and in promoting tumors might not exist. Rather, the pro-tumorigenic activity of CAFs may evolve gradually (see poster). It is, however, conceivable that the kinetics of such changes in CAF action(s) might be different in different tumor types, in part because the resident fibroblasts exhibit different organ-specific transcriptomic profiles (Rinn et al., 2006). Even within an individual tumor type, for example, in pancreatic cancer, different subtypes of CAFs can exert distinct paracrine actions (Box 1) that could impact tumor-enhancing inflammation (Öhlund et al., 2017).

The activation of fibroblasts was initially studied in the context of wound healing (Bainbridge, 2013; Klingberg et al., 2013). When damage occurs in normal tissue, the damaged epithelial cells and the immune cells recruited to the damage site release chemical mediators that initiate the activation of resident fibroblasts. These include damage-associated molecular patterns, as well as secreted growth factors (e.g. TGFβ proteins, PDGFs, FGF2; Box 2) and cytokines [INFγ (IFNG), TNFα (TNF), interleukins; Box 2] (Calon et al., 2014; Rasanen and Vaheri, 2010) (see poster). With respect to CAFs, the transition from quiescent fibroblasts to activated CAFs might depend on additional chemical mediators, including growth factors, cytokines and metabolites aberrantly produced by the malignant cells and by the recruited immune cells (Harper and Sainson, 2014; Kalluri, 2016; Roy and Bera, 2016). As mentioned above, the activated state of CAFs requires metabolic reprogramming (Martinez-Outschoorn et al., 2014; Roy and Bera, 2016; Wu et al., 2017; Yang et al., 2016; Zhang et al., 2015), presumably to enable their enhanced proliferation and increased biosynthetic functions, such as the production of extracellular proteins like collagens, laminins, elastin and others (Alexander and Cukierman, 2016).

It is often presumed that in a growing tumor, CAFs are the dominant producer of ECM proteins, in part reflecting the close proximity of CAFs to the areas of ECM remodeling (Alexander and Cukierman, 2016; Kalluri, 2016; Lu et al., 2011). However, emerging evidence suggests that the cancer cells themselves might also produce ECM components (Ozdemir et al., 2014), and acquired features of cancer cells, such as the loss of TGFβ signaling, specifically result in increased ECM production (Laklai et al., 2016). The desmoplastic reaction (Box 1) and accumulation of CAFs is often associated with cancer progression (Kalluri, 2016). Upon histological evaluation, such an abundance of CAFs and ECM in a tumor specimen might, however, simply reflect a more advanced stage of tumor progression, rather than being causally associated with a poor clinical outcome.

Several studies attempted to identify activated CAFs by examining a number of biological markers and transcriptional changes, with a number of groups attempting to characterize specific CAF markers. But, as discussed below, these attempts were rarely successful. The markers that are most commonly used to identify CAFs in *in vivo* pre-clinical and in clinical studies (reviewed in Cortez et al., 2014; Criscitiello et al., 2014; Hematti, 2012; Kalluri, 2016; Madar et al., 2013; Marsh et al., 2013; Rasanen and Vaheri, 2010; Shiga et al., 2015) include (see poster and Box 2):

(1) ECM components, such as collagen I, collagen II, fibronectin, tenascin C (TN-C) and periostin, and remodeling enzymes, such as LOX, LOXL1, MMPs and TIMPs (De Wever et al., 2004; O'Connell et al., 2011);
(2) growth factors and cytokines, such as TGFβs, VEGFs, PDGFs, EGF, FGFs, PGE2, CTGF, SDF-1 (CXCL12) and WNTs (Erez et al., 2010; O'Connell et al., 2011; Orimo et al., 2005);
(3) receptors and other membrane-bound proteins, such as PDGFRα/β, VCAM1, DDR2, TGFβRI/II, EGFR, FGFRs, BMPRI (BMPR1A/B)/BMPRII, podoplanin and FAP, and a decreased expression of CAV1 (Quail and Joyce, 2013; Rasanen and Vaheri, 2010; Sotgia et al., 2009);
(4) cytoskeleton components and other cytoplasmic proteins, such as desmin, vimentin, αSMA and FSP1/S100A4 (Quail and Joyce, 2013; Sugimoto et al., 2006).

The heterogeneity of such markers in distinct tumor types (Cortez et al., 2014; Sugimoto et al., 2006), and expression of some of these markers in normal tissues (Council and Hameed, 2009), pose a significant challenge when studying the role of CAFs and their biological properties in cancer. For example, distinct overlap in FSP-1/S100A4 and αSMA expression in CAFs from breast tumor compared with pancreatic tumors (as detailed above, Sugimoto et al., 2006) add an additional level of complexity when attributing functions of CAFs defined by either of these individual CAF markers in a given tumor type. In addition, analyzing the signaling pathways that occur in CAFs as opposed to the malignant cells in the tumor is challenging, because receptors such as PDGFRα/β, TGFβRI/II, EGFR, FGFR, BMPRI/II and others can be expressed by both CAFs and the malignant cells. Therefore, it is likely that studying CAFs will require the use of multiple identifying markers in parallel.

That said, genetically engineered mouse models (GEMMs) are offering new insights on the functional heterogeneity of CAFs, including the definition of CAF markers in relation to their function in the tumors (O'Connell et al., 2011; Ozdemir et al., 2014; Rhim et al., 2014). For example, the study of GEMMs designed to limit the accumulation of CAFs in growing pancreatic tumors (Ozdemir et al., 2014), or to conditionally delete the pro-angiogenic growth factor VEGFs in breast CAFs (O'Connell et al., 2011), revealed that there are distinct functional subtypes of CAFs. Additionally, the use of defined gene promoter-driven expression of viral thymidine kinase (TK) proteins in GEMMs to study CAFs has enabled researchers to deplete distinct populations of proliferating CAFs using ganciclovir, a compound that is only toxic to cells that express viral TK. This system is described in Cooke et al. (2012), LeBleu et al. (2013) and O'Connell et al. (2011), and is being actively used to determine the functions of CAFs in various tissues. For example, in breast cancer, the ganciclovir-mediated depletion of proliferating FSP1/S100A4^+^ stromal cells did not impact primary tumor growth, but it resulted in suppressed metastasis (O'Connell et al., 2011). In this context, it is possible that FSP1/S100A4^+^ cells promoted metastatic disease via the secretion of VEGFA and TN-C, which remodel blood vessels and can provide protection from apoptosis, respectively (O'Connell et al., 2011). In contrast, a similar approach used to deplete CAFs expressing αSMA, a dominant CAF population in the pancreatic desmoplastic reaction, suggested that αSMA^+^ stromal cells were predominantly acting to restrain, rather than to promote, cancer progression. Thus, their depletion resulted in more aggressive tumors, suggesting that αSMA^+^ CAFs might play a role in controlling the tumor immune response and that their depletion results in a more immunosuppressive tumor microenvironment (Ozdemir et al., 2014). Although more studies are needed, these results support the hypothesis that distinct CAFs, as defined by their expression of specific markers, exert either anti- or pro-tumor functions, and that these might also be tumor type dependent.

## The pro- and antitumor functions of CAFs

As indicated by the GEMM studies discussed above, distinct subsets of CAFs present with cancer-restraining or cancer-promoting functions. The interplay of CAFs and cancer cells within the TME can be depicted as a highly complex signaling network, with dynamic axes of signaling that can oppose or synergize to influence each other's function and impact on cancer progression and metastasis (Gascard and Tlsty, 2016; Ishii et al., 2015; Kalluri, 2016; Luo et al., 2015; Marsh et al., 2013; Mezawa and Orimo, 2016).

Early studies using ad-mixing experiments, wherein cultured CAFs and cancer cells were mixed together prior to their injection in mice, largely investigated the pro-tumorigenic influence of CAFs on cancer cells. This work supports the notion that CAFs have pro-tumorigenic effects, as indicated by the more aggressive formation of tumors in mice or enhanced proliferation or migration of cancer cells *in vitro* (Berdiel-Acer et al., 2014; Erez et al., 2010; Karnoub et al., 2007; Orimo et al., 2005; Tyan et al., 2011). However, fibroblasts are often referred to as ‘easy to culture’ and are indeed a cell type that has demonstrated robust adaptation to *ex vivo* expansion on plastic (see poster). Tumor-promoting CAFs, secreting pro-survival factors, might have a selective advantage over tumor-restraining CAFs when propagated *in vitro*. This could thus have biased ad-mixing studies in which tumor cells were selectively mixed with a CAF population that became enriched for their tumor-promoting properties. Thus, the interpretation of early ad-mixing studies should consider the possibility of a preferential culture enrichment of pro-tumorigenic CAFs (Kalluri, 2016).

Activated fibroblasts, which have similar features to mesenchymal stromal cells (MSCs, Box 1) (Hematti, 2012), have been shown to possess intrinsic cellular plasticity, challenging their functional characterization as being capable of reprogramming into distinct lineages, including endothelial cells, adipocytes and chondrocytes (Gascard and Tlsty, 2016; Kalluri, 2016; Lorenz et al., 2008; Ubil et al., 2014). If the same is true for CAFs, such multi-lineage differentiation potential might then also be associated with a change in their tumor-promoting or -restraining functions. To discern the precise roles of CAFs in tumors, multiple approaches will be needed to overcome the experimental limitations in the systems studied, as well as to overcome the heterogeneity of CAF markers. The current experimental limitations include a lack of precise *in vivo* (mouse) modeling and imaging tools to dissect the molecular determinants of CAF functions during cancer progression, to track their heterogeneous marker expression over time, and to mechanistically probe their functional relationship with other components of the TME, such as the immune cell infiltrate, ECM, and intratumoral hypoxia and angiogenesis. Comparative analyses between tumor models and tumor types that would be aimed at determining the overlap (or lack thereof) of distinct CAF markers, used concomitantly with putative non-CAF markers and lineage tracing analyses, could help with the correct interpretation of existing studies, such as those cited in this Review, that remain limited by a lack of in-depth knowledge of the heterogeneous CAF markers. Further, the study of CAF functions will also need to consider the distinct stages of cancer progression, and a likely evolution of the co-dependency between CAFs and cancer cells in their dynamic microenvironment. The impact of CAFs on cancer progression is not limited to their direct influence on cancer cells, but also extends to other cellular components of the primary and metastatic lesions that regulate tumor-mediated reprogramming of the vasculature and of the immune system (Barnas et al., 2010; Erez et al., 2010; Fukumura et al., 1998; Guo et al., 2008; Gyotoku et al., 2001; Liao et al., 2009; Raz and Erez, 2013; Tang et al., 2016). The complexity of the functional relationships of CAFs to cancer cells and other cellular populations in the TME further implicates that CAFs can serve as both tumor-promoting and tumor-restraining entities during cancer progression: for example, a given population of CAFs exerts tumor-promoting functions onto cancer cells, but can exert tumor-restraining functions by remodeling the TME (Augsten, 2014; Gascard and Tlsty, 2016; Han et al., 2015; Harper and Sainson, 2014; Mezawa and Orimo, 2016). A more precise understanding of the overall implications of CAFs in relation to multiple components of the TME, as well as to cancer cells, could enable a better future therapeutic design to limit tumor-promoting CAFs functions while enhancing their tumor-restraining functions.

### Pro-tumorigenic functions of CAFs

The pro-tumorigenic functions of CAFs (see poster) are generally driven by their altered secretome (Erez et al., 2010; Mezawa and Orimo, 2016; Orimo et al., 2005; Raz and Erez, 2013). The paracrine signaling between CAFs and cancer cells, wherein CAFs secrete growth factors and cytokines such as CXCL12 (Orimo et al., 2005; Yu et al., 2014b), CCL7 (Jung et al., 2010), TGFβs (Calon et al., 2014; Yu et al., 2014b; Zhuang et al., 2015), FGFs (Bai et al., 2015; Henriksson et al., 2011; Sun et al., 2017), HGF (De Wever et al., 2004; Jedeszko et al., 2009; Tyan et al., 2011), periostin (POSTN) (Kikuchi et al., 2008; Ratajczak-Wielgomas et al., 2016) and TN-C (De Wever et al., 2004; O'Connell et al., 2011), might directly and positively impact tumor progression by enhancing the survival, proliferation, stemness, and the metastasis-initiating capacity of cancer cells, ultimately promoting cancer progression, but also enhancing resistance to therapy. In light of these studies, the paracrine signaling between CAFs and cancer cells has been characterized as a reciprocal and convergent set of signaling activities that promote tumor growth and cancer invasion and metastasis (Alexander and Cukierman, 2016; Cirri and Chiarugi, 2012; De Wever et al., 2014; Han et al., 2015; Mezawa and Orimo, 2016). CAFs are also effective in the remodeling of the tumor vasculature through the secretion of VEGFs, FGFs and IL-6, and of the ECM through the secretion of MMPs and ECM proteins, and in modulating pro-tumorigenic inflammation through the secretion of IL-1 (IL1A), IL-6, TNFα, TGFβs, SDF-1 and MCP-1 (CCL2). These represent the indirect influences of CAFs in promoting tumor growth, wherein the CAF secretome enhances angiogenesis and ECM stiffness to promote the survival, proliferation and migration of cancer cells, and generates an immunosuppressive microenvironment that limits antitumor immunity (reviewed in Gascard and Tlsty, 2016; Han et al., 2015; Harper and Sainson, 2014; Kalluri, 2016; Marsh et al., 2013; Raz and Erez, 2013). CAFs were also reported to exert a physical force, transmitted by CAF-cancer cell adhesion, to promote a cooperative collective invasion or co-migration of CAFs and cancer cells (Labernadie et al., 2017), supporting the notion that a direct cell-cell contact between CAFs and cancer cells promotes cancer cell invasion.

The tumor immunity and the intratumoral vascular program are regulated by cytokines and chemokines that are secreted by CAFs (Erez et al., 2010; Fukumura et al., 1998; Guo et al., 2008; Liao et al., 2009; Tang et al., 2016). However, a mechanistic understanding of how CAFs co-regulate their own signaling network with the signaling networks of immune cells and blood vessels will require more studies. Indeed, many of the CAF-derived chemokines and cytokines that were mentioned above also function in a positive feedback loop to enhance or perpetuate CAF activation (Kalluri, 2016; Rasanen and Vaheri, 2010). Furthermore, whether cancer cells directly influence the CAF secretome to promote tumor growth remains to be determined with further *in vivo* functional studies. It is conceivable that the tumor-promoting functions of CAFs are due to ‘collateral damage’ from their otherwise protective, wound repair activities, and that cancer cells merely benefit from a CAF secretome that was originally intended for wound repair. We postulate that this could possibly occur in the early stages of oncogenesis, which might then be followed by a cancer cell-mediated reprogramming of CAFs to enhance tumor progression and facilitate metastasis. For example, the pro-inflammatory cytokine LIF (Box 2), secreted by both CAFs and cancer cells, was found to mediate the epigenetic modifications of CAFs in order to enhance their pro-tumorigenic functions, namely by enhancing the CAF acto-myosin contractility that enabled the CAFs to form ECM tracks, which were then used by the cancer cells in a collective invasion (Albrengues et al., 2015).

Finally, the pro-tumorigenic functions of CAFs could be attributed to their role in reprogramming and shaping the metabolic microenvironment of tumors (Kalluri, 2016; Lisanti et al., 2013) (see poster). Several lines of investigation support that metabolites, such as lactate and ketone bodies, are produced by CAFs and can support the growth and proliferation of the cancer (Martinez-Outschoorn et al., 2014; Yang et al., 2016) and the immune cells in the TME, specifically T cells (Ghesquiere et al., 2014; Molon et al., 2016).

### Antitumor functions of CAFs

While the pro-tumorigenic functions of CAFs are likely to be based on their production of pro-survival factors, which in turn enhance cancer cell proliferation and metabolic adaptation, their antitumor properties are predominantly associated with their functions as regulators of antitumor immunity (Kalluri, 2016) (see poster). Some of the clinical efforts to target CAFs, supported by preclinical studies, have offered novel insights into the heterogeneous function(s) of CAFs in cancer progression, and in some cases, as discussed in detail below, highlighted their antitumor properties (Öhlund et al., 2014, 2017; Ozdemir et al., 2014; Rhim et al., 2014). The depletion of CAFs using genetic strategies in GEMMs of pancreatic cancer revealed that proliferating αSMA-expressing CAFs do limit tumor progression rather than promoting it. Their depletion yielded a more invasive tumor with enhanced intratumoral hypoxia, as well as increased proportions of regulatory T cells (Ozdemir et al., 2014). A reduction in CAFs in GEMMs of pancreatic tumors harboring a genetic deletion of SHH (Box 2) in the cancer cells also resulted in more aggressive tumors with increased cancer cell proliferation, which was possibly mediated by an enhanced tumor vascularity (Rhim et al., 2014). Notably, in patient-derived pancreatic cancer samples, the abundance of αSMA^+^ CAFs did not correlate with a diminished intratumoral T cell infiltration, suggesting that these CAFs might promote T cell accumulation in the proximity of cancer cells *in vivo* (Carstens et al., 2017). Indeed, the CAF secretome might also exert antitumor functions; for instance, IL-10, TGFβs, IFNγ and IL-6 participate in the recruitment and polarization of macrophages, NK cells and T cells, which promote an immune control of cancer cells (reviewed in Harper and Sainson, 2014; Kalluri, 2016). Thus, the net effect of the CAF secretome must be considered as bimodal and dynamic. The use of GEMMs and sophisticated experimental methodologies to determine the functional heterogeneity of CAFs, thereby linking defined cellular markers to specific CAF functions, will help to further discern the pro- and antitumor functions of CAFs in distinct tumor types.

## Therapeutic targeting of CAFs

The development of anticancer therapies to target CAFs has largely focused on their pro-tumorigenic functions. Most conventional anticancer therapeutic approaches are likely to affect CAFs as well, because highly proliferating cells are more sensitive to agents that affect generic signaling networks, induce DNA damage, impede DNA/RNA synthesis and block the cytoskeletal remodeling necessary for cell division. Although the potency of chemo- and radiotherapy is based on the premise that cancer cells will have enhanced sensitivity, as they are more proliferative, the unintended impact of such therapeutic interventions on the function or accumulation of CAFs is largely unknown. Depletion of FAP^+^ cells using genetic strategies resulted in a cachexia and anemia phenotype in mice (Roberts et al., 2013), underscoring that strategies to target CAFs for anticancer therapies must also take into consideration the systemic side effects, such as the risk of developing cachexia, anemia and other paraneoplastic syndromes. Nonetheless, depletion of FAP^+^ CAFs in mice with pancreatic cancer enabled the antitumor efficacy of immune checkpoint blockade, namely anti-CTLA4 and anti-PD-L1 (CD274) antibodies (Box 2) (Feig et al., 2013). Depleting FAP^+^ CAFs in mice with melanoma also reduced the activity of immunosuppressive cells and improved antitumor activity of CD8^+^ tumor-infiltrating T cells (Zhang and Ertl, 2016). Although these studies support a functional role of FAP^+^ cells in immunosurveillance, the targeting of FAP^+^ CAFs, via adoptive transfer of FAP-targeted chimeric antigen receptor (CAR) T cells, can also suppress pancreatic cancer growth in mice by suppressing tumor angiogenesis (Lo et al., 2015).

CAFs have been implicated in promoting resistance to therapy, so there is an interest in devising a targeted anti-CAF therapeutic approach (Hale et al., 2013) (see poster). The cancer therapies currently used in the clinic can activate or modulate CAF functions. For example, targeting BRAF in melanoma was reported to activate CAFs to remodel the tumor ECM, thereby providing pro-tumorigenic signals that supported residual disease (Hirata et al., 2015). Furthermore, genotoxic stress and the associated damage induced by chemotherapeutic agents (e.g. mitoxantrone) caused transcriptomic changes in the CAFs, resulting in the secretion of WNT16B (WNT16), which signals to enhance survival and EMT in prostate cancer cells (Sun et al., 2012). There is also evidence that the CAF secretome and their ECM-remodeling properties could mediate resistance to chemotherapy by promoting invasion and dissemination of cancer cells via ECM degradation and vascular remodeling (reviewed in Kalluri, 2016; Kharaishvili et al., 2014). Resistance to chemotherapy could also be mediated by direct CAF-cancer cell signaling that promotes cancer cell survival when exposed to the cytotoxic effects of the chemotherapeutic agent cisplatin (Li et al., 2001). A recent study by Su et al. identified that CD10^+^ (MME^+^) GPR77^+^ (C5AR2^+^) CAFs promote breast cancer stem cell survival and resistance to chemotherapy through secretion of IL-6 and IL-8 (CXCL8) (Su et al., 2018). Although these findings support a role for CAFs in chemoresistance, the likely functional heterogeneity of CAFs, as discussed above, means that researchers should exercise caution when generalizing their pro-tumorigenic actions in the context of drug resistance studies (Kharaishvili et al., 2014; Öhlund et al., 2014).

A more effective approach to target CAFs could lie in delineating the regulatory pathways that lead to the activation of fibroblasts. In pancreatic cancer, the vitamin D analog calcipotriol was capable of reprogramming the CAFs to acquire the nonactivated phenotype of pancreatic stellate cells, the resident mesenchymal cells of the pancreas (Sherman et al., 2014). Clinical trials are ongoing to test whether such CAF reprogramming enhances the efficacy of gemcitabine, a chemotherapeutic drug used commonly in pancreatic cancer. Moreover, using JQ1, an inhibitor of the BET family of bromodomain chromatin-modulating proteins, in patient-derived xenografts of pancreatic cancer resulted in reduced activation of CAFs and attenuated tumor growth (Yamamoto et al., 2016). Finally, although conventional maximum-tolerated dose treatment is known to activate CAFs, applying metronomic chemotherapy (Box 1) was recently reported to limit such chemotherapy-induced activation of CAFs. Although maximum-tolerated dose chemotherapy enhanced CAF pro-tumorigenic functions, metronomic chemotherapy restricted the CAF pro-tumorigenic functions by decreasing the expression of chemokines, thereby limiting the expansion of the stem-like tumor-initiating cells following therapy (Chan et al., 2016).

The approaches summarized here will not only inform on the impact of targeting CAFs during cancer therapy, but can also provide additional insights into the biology of this important player of the TME. These novel insights could, in turn, impact novel and promising therapies, including future combination strategies that also aim to remodel the TME, such as antiangiogenic therapy and immunotherapy.

## Conclusions

The next decade will likely bring about many more discoveries regarding the biology of CAFs, informed by the development of new experimental tools that could more precisely define their functional contribution to cancer progression and therapy. Ongoing and future studies, employing novel approaches to monitor and functionally alter CAFs *in vivo*, will likely unravel new regulatory pathways involving CAFs in cancer progression. The precise definition of the heterogeneous CAF populations at distinct stages of cancer progression, with markers that inform on their functions, remains the most challenging aspect in the study of CAFs. Building on the precise knowledge of CAF markers to elucidate which CAF subpopulations exert a pro- versus antitumor effect will likely be beneficial for cancer treatment. Results from such studies could ultimately offer insights into novel combination therapies aimed at exploiting the therapeutic vulnerabilities of the TME, and at reprogramming the CAFs and other components of the TME to control cancer progression and enable efficient therapeutic responses.
